# CircKIF4A promotes non-small cell lung cancer proliferation and metastasis through MiR-1238/CLDN14 axis

**DOI:** 10.18632/aging.204276

**Published:** 2022-09-12

**Authors:** Weimin Luo, Yuefeng Liu, Hangying Qu, Xiangyu Luo, Liqiang Xu, Jia Zhang, Jiansheng Wang

**Affiliations:** 1 Department of Thoracic Surgery, The First Affiliated Hospital of Xi'an Jiaotong University, Xi'an, Shaanxi 710061, China; 2 Department of Ophthalmology Surgery, Taihe Hospital, Hubei University of Medicine, Shiyan, Hubei 442000, China; 3 Department of Cardiothoracic Surgery, Taihe Hospital, Hubei University of Medicine, Shiyan, Hubei 442000, China

**Keywords:** circKIF4A, non-small cell lung cancer, miR-1238, CLDN14, metastasis

## Abstract

As the leading cause of cancer-related death worldwide, non-small-cell lung cancer (NSCLC) is still in need of improved therapeutic strategies. CircKIF4A has been found to be involved in the progression of multiple cancers while its role in NSCLC remains unclear. To investigate the functions of circKIF4A, we assessed the expression of circKIF4A in NSCLC cells and tissues and performed experiments to determine the detailed functions of circKIF4A in NSCLC, including migration and proliferation. We found CircKIF4A expressed more heavily in the cells and tissues of NSCLC patients, and functional studies showed that inhibition of circKIF4A reduced NSCLC cells metastasis and proliferation. Furthermore, we seek to identify the underlying regulatory effect of circKIF4A in NSCLC. Studies revealed that circKIF4A sponged miR-1238 to promote NSCLC progression by up-regulating claudin14 (CLDN14) expression. In conclusion, circKIF4A is a potential diagnostic and therapeutic target in the circKIF4A/miR-1238/CLDN14 axis that plays an important role in NSCLC progression.

## INTRODUCTION

Lung cancer is still major burden of death around the world, mainly because of high metastasis rate [1]. Among of all lung cancers, non-small-cell lung cancer (NSCLC) occupies nearly 85%. Despite improvements in overall survival of patients by a variety of treatment strategies especially targeted therapy and immunotherapy, NSCLC still has a bleak prognosis. In order to develop novel and useful therapeutic tools to better control this disease, a further understanding of the mechanisms of NSCLC progression is urgently needed.

Deregulation of non-coding RNAs (ncRNAs) has been reported involved in lung cancer progressions [2, 3]. CircRNAs play key roles in lung cancer tumorigenesis, progress, invasion and metastasis and might be promising diagnosis and therapeutic targets [4, 5].

Previous studies have found that circKIF4A (hsa_circ_0007255) is vital in multiple cancer progressions. In ovarian cancer, circKIF4A was highly expressed and enhanced cell proliferation and migration [6]. In glioma, circKIF4A promoted tumor development through the miR-139-3p/Wnt5a axis [7]. Besides, circKIF4A regulated KIF4A expression to affect TNBC progression via sponging miR-375 [8]. However, it remains unclear how circKIF4A impacts upon NSCLC.

In this study, circKIF4A was found over-expressed in NSCLC cell lines and tissues. Besides, inhibition of circKIF4A decreased NSCLC metastasis and cell proliferation. CircKIF4A was found sponged miR-1238 to increase the expression of CLDN14, which promotes NSCLC progression. CircKIF4A might be an underlying therapeutic target for NSCLC treatment.

## RESULTS

### circKIF4A is upregulated in NSCLC

**Figure 1:**
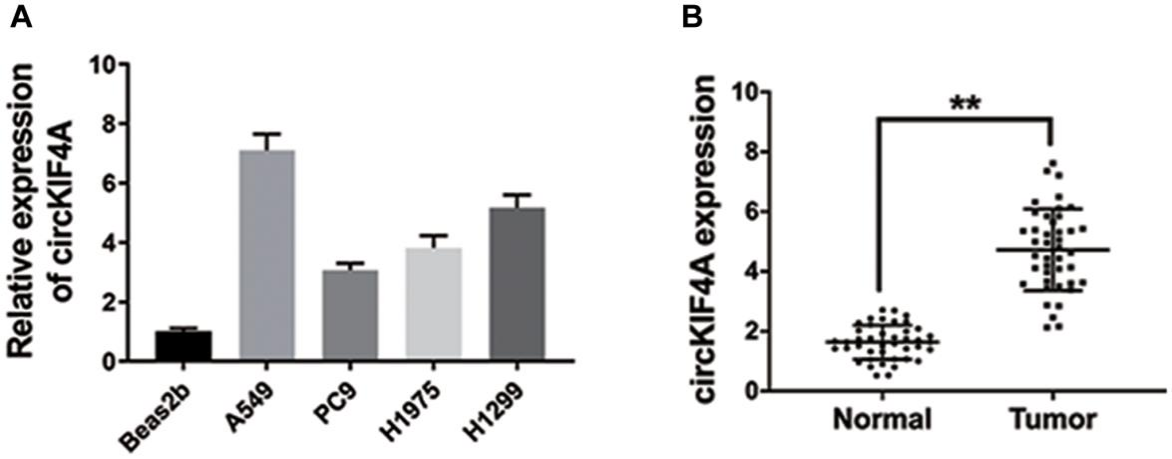
**circKIF4A is upregulated in NSCLC.** (**A**) The circKIF4A expression in NSCLC cell lines. (**B**) The expression of circKIF4A in 42 pairs of NSCLC tissues and adjacent normal tissues. ^**^P < 0.01.

We performed qRT-PCR in NSCLC cell lines. Figure 1A showed that it over-expressed compared with normal cell line. Next, we collected 42 pairs of NSCLC tissues and normal tissues. The results further confirmed our conclusion (Figure 1B).

### Knockdown of circKIF4A suppresses NSCLC cell proliferation and metastasis

**Figure 2:**
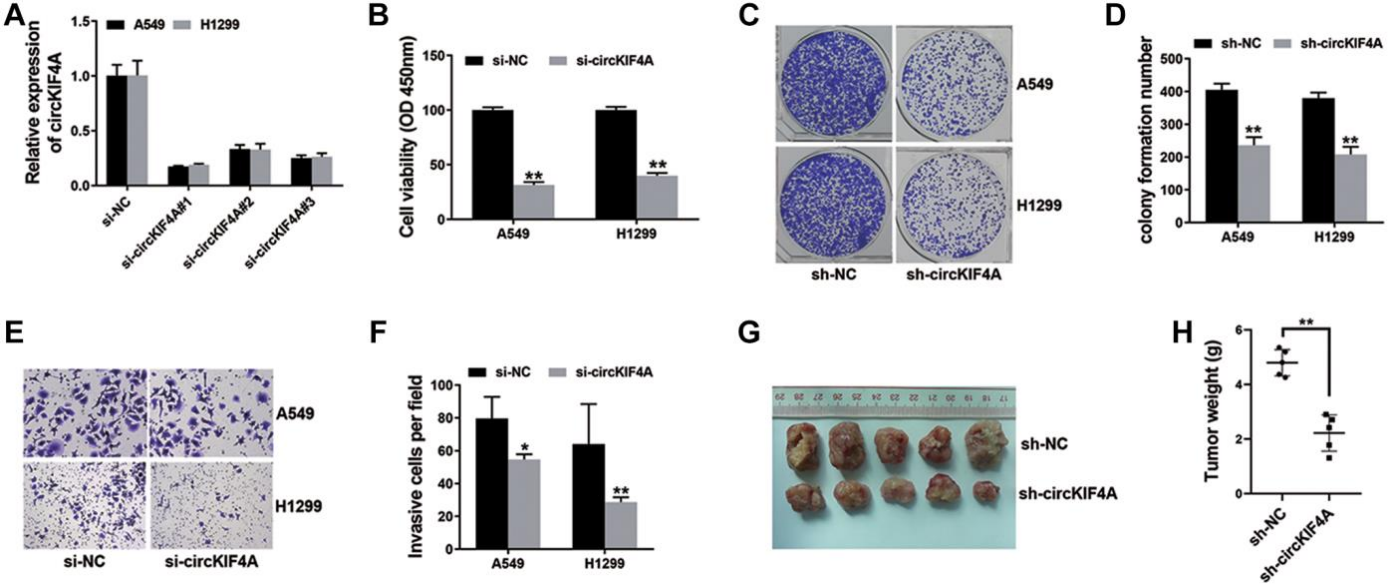
Knockdown of circKIF4A suppresses NSCLC cell proliferation and metastasis (^*^P < 0.05, ^**^P < 0.01). (**A**) siRNAs were used to knockdown circKIF4A expression. (**B**) CCK-8 assay was performed to investigate cell proliferation ability. (**C**) Colony formation assay was conducted to reveal cell colony-forming ability. (**D**) Barplot drawn by ImageJ. (**E**) The result of transwell assay. (**F**) Barplot drawn by ImageJ. (**G**) Mouse xenograft models were used to evaluate circKIF4A function *in vivo*. (**H**) Result of xenograft tumor weights.

We used siRNAs to knockdown circKIF4A and si-circKIF4A#1 was chosen for the further experiments (Figure 2A). The result of CCK-8 assay showed that the inhibition of circKIF4A suppressed cell proliferation (Figure 2B). Besides, circKIF4A inhibition restrained the colony formation ability of NSCLC cells (Figure 2C, 2D). Additionally, transwell assay indicated that knockdown of circKIF4A decreased NSCLC cells metastasis (Figure 2E, 2F). Finally, we used xenograft models to explore the functions of circKIF4A. We found that knockdown of circKIF4A suppressed tumor growth in NSCLC (Figure 2G, 2H).

### circKIF4A performs as a sponge for miR-1238

**Figure 3:**
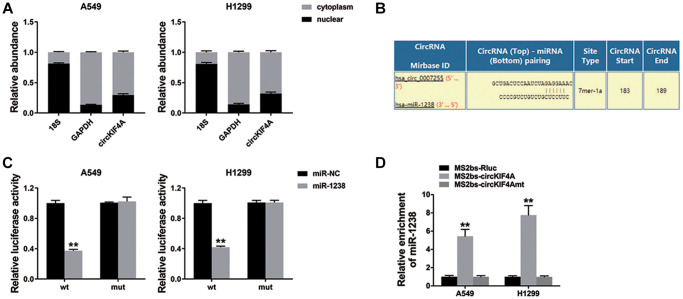
circKIF4A performs as a sponge for miR-1238 (^**^P < 0.01). (**A**) qRT-PCR detected the levels of GAPDH, 18S, and circKIF4A. (**B**) The potential miR-1238 binding sites within circKIF4A. (**C**) The result of Luciferase assay. (**D**) The result of MS2-based RIP assay.

circRNAs could regulate gene expression by acting as microRNA decoys. Here, we explored the sub-location of circKIF4A in NSCLC cells. Figure 3A revealed circKIF4A was mostly located in cell cytoplasm. Next, Circular RNA Interactome was devoted. Figure 3B showed the predicted interaction and binding sites for miR-1238 in circKIF4A sequence. Therefore, luciferase reporter assay was conducted and the result showed that the co-transfected cells with wild type luciferase reporter and miR-1238 mimics led to the reduction of luciferase intensity (Figure 3C). We also performed RIP assay and found that MS2bs-circKIF4A group was enriched in miR-1238, suggesting that circKIF4A could directly bind with miR-1238 to sponge miR-1238 (Figure 3D).

### miR-1238 suppresses NSCLC cell proliferation and metastasis

**Figure 4:**
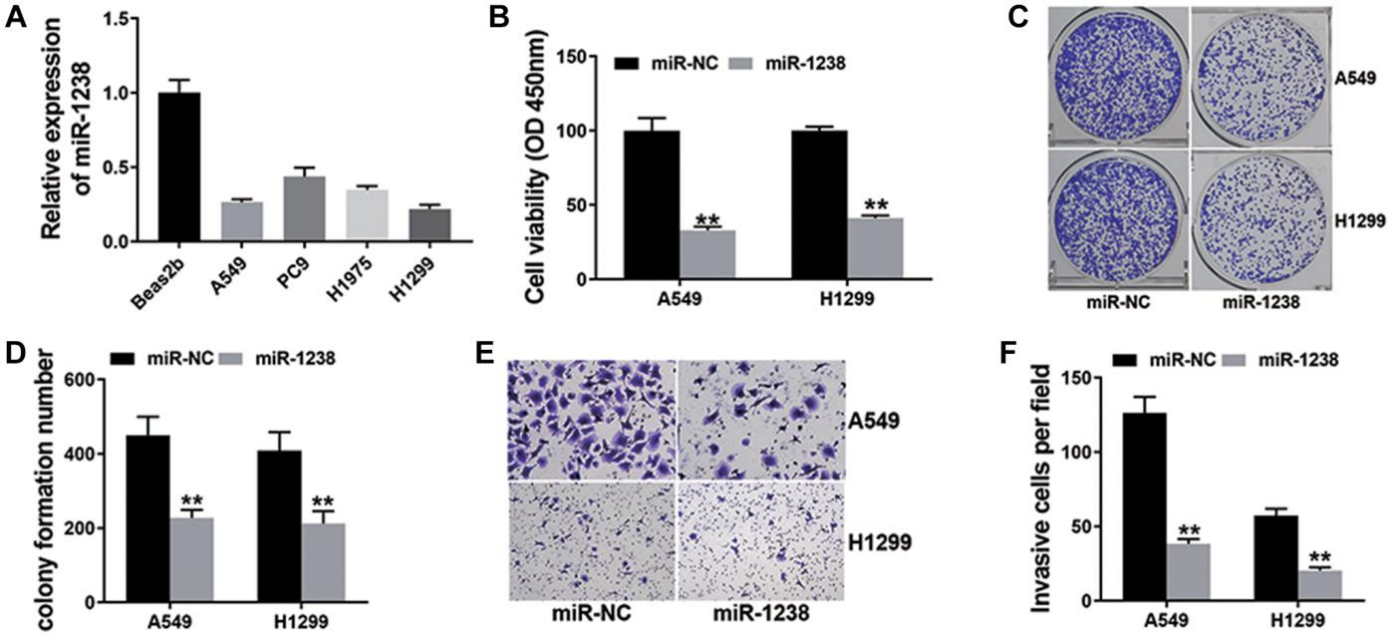
miR-1238 suppresses NSCLC cell proliferation and metastasis (^**^P < 0.01). (**A**) qRT-PCR detected miR-1238 expression in NSCLC cell lines. (**B**) CCK-8 assay was performed to investigate cell proliferation ability. (**C**) Colony formation assay was conducted to reveal cell colony-forming ability. (**D**) Barplot drawn by ImageJ. (**E**) Transwell assay was performed to detect cell metastasis. (**F**) Barplot drawn by ImageJ.

Next, we assessed miR-1238 expression in NSCLC cell lines and the result showed miR-1238 was down-regulated (Figure 4A). We conducted CCK-8 assay and found that miR-1238 could suppress NSCLC cell proliferation (Figure 4B). Additionally, we observed that miR-1238 suppressed the colony formation ability of NSCLC cells (Figure 4C, 4D). Finally, we conducted transwell assay and found that miR-1238 decreased the metastasis ability of NSCLC cells (Figure 4E, 4F).

### circKIF4A functions as a ceRNA to regulate CLDN14

**Figure 5:**
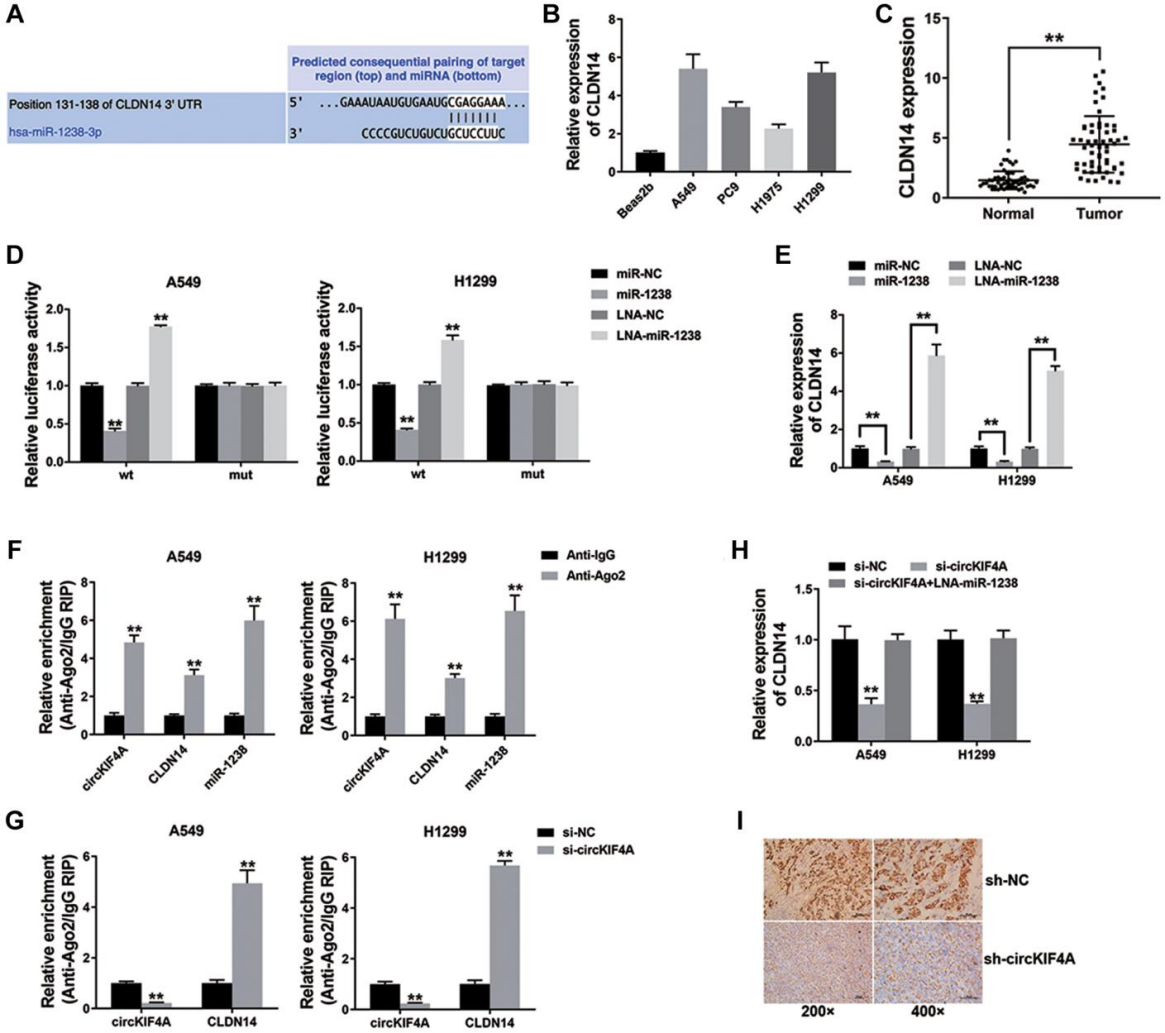
circKIF4A functions as a ceRNA to regulate CLDN14 (^**^P < 0.01). (**A**) The predicted miR-1238 binding sites within CLDN14. (**B**) qRT-PCR detected CLDN14 expression in NSCLC cell lines. (**C**) CLDN14 expression in 42 pairs of normal adjacent tissues and NSCLC tissue. (**D**) The result of Luciferase assay. (**E**) qRT-PCR detected CLDN14 expression. (**F**) Enrichment of circKIF4A, CLDN14 and miR-1238 to Ago2. (**G**) RIP assay on Ago2 was performed. (**H**) qRT-PCR detected CLDN14 expression. (**I**) IHC staining of CLDN14 were shown.

We searched TargetScan to explore if circKIF4A sponges miR-1238 to regulate downstream target, and claudin14 (CLDN14) was predicted (Figure 5A). Next, we explored CLDN14 expression in NSCLC cell lines and tissues and CLDN14 was up-regulated (Figure 5B, 5C). Luciferase reporter assay revealed that co-transfection with miR-1238 mimics and wild type luciferase reporter decreased the intensity of luciferase, while co-transfection with miR-1238 inhibitor and wild type luciferase reporter increased (Figure 5D). Figure 5E showed that miR-1238 could reduce CLDN14 expression, and miR-1238 inhibitor had a opposite effect on CLDN14.

We conducted Subsequent RIP assay on Ago2 and found that circKIF4A, CLDN14, and miR-1238 were basically concentrated in Ago2 (Figure 5F). Moreover, the result of Figure 5G showed circKIF4A functioned as a ceRNA to compete with CLDN14 for binding miRNAs. Moreover, inhibition of circKIF4A lowered the expression of CLDN14, which was contrary to the result of co-transfection with miR-1238 inhibitor. The result of Figure 5H indicated that circKIF4A sponged miR-1238 to regulate CLDN14 expression in NSCLC. Finally, CLDN14 expression in mouse xenograft models has been measured, the result showed that circKIF4A inhibition lowered the expression of CLDN14 *in vivo* (Figure 5I).

## DISCUSSION

NSCLC remains major burden worldwide [9]. Despite various treatments have occurred, the mortality rate remains high [10]. There are still many problems to be solved urgently. Exploring the underlying mechanisms of NSCLC proliferation and metastasis could help develop individual therapeutic strategies.

Non-coding RNAs are associated with lung cancer progressions and could serve as predictive biomarkers [11]. CircRNAs are vital in NSCLC generation and progression [12, 13]. For instance, circRNA_102481 contributed to EGFR-TKIs resistance via the miR-30a-5p/ROR1 axis, which could be an underlying target in NSCLC [14].

CircKIF4A is a promotor in multiple cancers. CircKIF4A facilitated tumor malignant progress via the miR-1231/GPX4 axis in papillary thyroid cancer [15]. CircKIF4A sponged miR-375/1231 accelerates tumor progression via up-regulating NOTCH2 expression in bladder cancer [16]. Besides, circKIF4A promoted metastasis and reduced cell apoptosis by miR-152/ ZEB1 axis in breast cancer [17]. However, it has not been reported that how circKIF4A functions in NSCLC.

Here, we assessed circKIF4A expression of NSCLC cell lines (Figure 1). Moreover, inhibition of circKIF4A caused the suppression of proliferation and metastasis, suggesting a vital role of circKIF4A in NSCLC progression (Figure 2).

miR-1238 is reported as a suppressor in multiple cancers [18–20]. CircRNAs has the ability to sponge miR-1238 to enhance tumor progressions. circ0070934 promotes cell metastasis by sponging miR-1238/1247-5p in cutaneous squamous cell carcinoma, [21]. In osteosarcoma, circular RNA circ_0000502 accelerates cell proliferation and invasion via sponging miR-1238 [22]. In NSCLC, miR-1238 also suppressed tumor cells by targeting LHX2 [23]. Besides, the results also indicated that miR-1238 was lower expressed in NSCLC cell lines, and miR-1238 suppressed NSCLC cell proliferation and metastasis (Figure 4). Besides, circKIF4A could combine miR-1238 and serve as a sponge for miR-1238 (Figure 3).

It has been confirmed that Claudins (CLDNs) were up-regulated in multiple cancers [24]. Among them, CLDN14 could promote tumor proliferation, and invasion through the PI3K/AKT/mTOR pathway [25]. CLDN14 was also found up-regulated in gastric cancer tissues and was related to E-cadherin expression and lymph node metastasis [26]. However, the functions of CLDN14 in NSCLC are still unclear. Here, we assessed CLDN14 expression and found it up-regulated in NSCLC. Acting as a downstream target, CLDN14 could be regulated by miR-1238. Further experiments showed that circKIF4A served as a ceRNA for miR-1238 to enhance CLDN14 expression of NSCLC (Figure 5).

In conclusion, we showed that the circKIF4A/miR-1238/CLDN14 axis was involved in NSCLC proliferation and metastasis. Targeting circKIF4A is promising for NSCLC treatment.

## MATERIALS AND METHODS

### Cell culture and transfection

Cell lines included Beas2b (normal lung cell line), A549, PC9, H1975 and H1299 (NSCLC cell lines). All of them were purchased from ATCC. DNA fingerprinting was performed to ensure cell authenticity. We also performed the detection for mycoplasma infection routinely.

The transfection was done with Lipofectamine 3000 (Invitrogen, USA). miR-1238 mimics and inhibitors, circKIF4A siRNAs, and circKIF4A shRNAs were purchased from GeneCopoeia (USA). Corresponding siRNA sequences of si-NC, si-circKIF4A#1, #2, and #3 were UUCUCCGAACGUGUCACGUTT, GCCUGGAUCUAUAACGUAUTT, GAUCUAUAACGUAUUAAUATT, and UAACGUAUUAAUAUUAACCTT, respectively.

### Quantitative real-time PCR (qRT-PCR) analysis

TRIzol (Invitrogen) was utilized to extract total cellular RNA. Cytoplasmic Extraction Reagents (Thermo Fisher Scientific, USA) and NE-PERTM Nuclear were used to extract nuclear and cytoplasmic RNA fractions. An All-in-OneTM miRNA qRT-PCR Detection Kit (GeneCopoeia) and SYBR Premix Ex TaqTM (Takara Bio, Japan) were applied to execute qRT-PCR assay. We synthesized the qRT-PCR primers by GeneCopoeia as follows: Forward of circKIF4A: GAGGTACCCTGCCTGGATCT; Reverse of circKIF4A: TGGAATCTCTGTAGGGCACA; Forward of 18S: TTAATTCCGATAACGAACGAGA; Reverse of 18S: CGCTGAGCCAGTCAGTGTAG; Forward of GAPDH: GGAGCGAGATCCCTCCAAAAT; Reverse of GAPDH: GGCTGTTGTCATACTTCTCATGG; Forward of CLDN14: AGCGGCATGAAGTTTGAGATT; Reverse of CLDN14: CCCGATTGTCTTTGTAGGCAG.

### Clinical sample collection

We collected 42 pairs of primary NSCLC and adjacent normal lung tissues from the First Affiliated Hospital, Xi’an Jiaotong University and immediately frozen into liquid nitrogen. We extracted and submitted total RNA to qRT-PCR analysis. We got approval of this study by the Ethics Committee of the First Affiliated Hospital, Xi’an Jiaotong University and performed based on the Declaration of Helsinki. All patients have provided written informed consents.

### Cell counting kit-8 assay (CCK-8)

We resuspended cells (1 × 10^3^) were and titrated into 96-well plates after transfection. Cells were incubated for 48 h at a temperature of 37°C before adding 10 μl CCK-8 solution (Dojindo Laboratories, Japan). We measured the absorbance at 450 nM after incubation for 2 h at 37°C, with microtiter plate reader (Bio-Tek EPOCH2, USA).

### Colony formation assay

We totally resuspended 1 × 10^3^ cells and seeded in 6-well plates. By 14-days incubation at 37°C, methanol fixed with colonies and stained with 0.1% crystal violet. We used ImageJ software to enumerate the colony number.

### Transwell assay

We resuspended a total of 1 × 10^4^ cells and seeded in the upper migration chambers (BD Biosciences, USA). Simultaneously, we added 10% FBS which was a chemoattractant to the lower chamber. The upper chambers were collected and the cells were further fixed with methanol after one day. Then, 0.1% crystal violet were applied for staining. The cells under the upper chamber were imaged and calculated by ImageJ software.

### Mouse xenograft model

We got approval of animal experiments and the experiments were performed following the guidelines of Institutional Animal Care and Use Committee of the First Affiliated Hospital, Xi’an Jiaotong University. We subcutaneously injected a total of 2 × 10^6^ A549 cells into 5 male nude mice (4-week-old). We excised the xenograft tumors with the condition of anesthesia to measure the weights of tumors.

### Luciferase reporter assay

The cells were seeded in 96-well plate with the amount of 3 × 10^4^ cells per well. Mutation was made in the predicted miR-1238 binding sites of circKIF4A and 3′-UTR of CLDN14. The miRNA mimics, inhibitors and constructed reporting vectors (circKIF4A-wt/mut or CLDN14 3′-UTR-wt/mut) were co-transfected into cells for 48 h. The relative luciferase signal was further detected using dual-luciferase reporter assay (Promega, USA).

### RNA immunoprecipitation (RIP)

We transfected cells with different treatment included MS2bs-Rluc, MS2bs-circKIF4A-mt, and MS2bs-circKIF4A. We used Magna RIP RNA-Binding Protein Immunoprecipitation Kit (Millipore, USA) to conduct RIP assay after incubating for 48 h. The expression of miR-1238 was assessed as RNA complexes purification.

We performed RIP assays for AGO2 with Millipore. Relative abundance of circKIF4A, CLDN14 and miR-1238 was measured later.

### Immunohistochemistry (IHC) analysis

The tissues on the slides were incubated in 3% H_2_O_2_ solution for 15 minutes after deparaffinization and rehydration at room temperature. The antigen retrieval was further performed using citrate buffer in a cooker at 96°C for 4 min. After blocking by goat serum, antibody against CLDN14 (dilution 1:200, Affinity, USA) were used for incubation overnight at 4°C. The slides were incubated at room temperature for 10 minutes with biotinylated secondary antibody, and finally HRP-Streptavidin. The slides were imaged after DAB staining.

### Statistical analysis

We conducted statistical analysis with SPSS 25.0. *T* tests were applied to make comparisons between groups. We set *P* < 0.05 as a significant value. Unless specific description, we presented data as the mean ± 3 S.D.
